# Ethical Challenges and Opportunities of AI in End-of-Life Palliative Care: Integrative Review

**DOI:** 10.2196/73517

**Published:** 2025-05-14

**Authors:** Abel García Abejas, David Geraldes Santos, Fabio Leite Costa, Aida Cordero Botejara, Helder Mota-Filipe, Àngels Salvador Vergés

**Affiliations:** 1 Faculty of Health Sciences University of Beira Interior Lisbon Portugal; 2 Palliative care Hospital Lusíadas Lisboa Lisbon Portugal; 3 Department of Communication, Philosophy and Politics University of Beira Interior Lisbon Portugal; 4 Pharmacology and Health Technologies Faculty of Pharmacy University of Lisbon Lisbon Portugal; 5 Board of Directors Innohealth.Academy Barcelona Spain

**Keywords:** palliative medicine, artificial intelligence (AI), ethical implications, end-of-life care, patient-centred care

## Abstract

**Background:**

Artificial intelligence (AI) is increasingly integrated into palliative medicine, offering opportunities to improve quality, efficiency, and patient-centeredness in end-of-life care. However, its use raises complex ethical issues, including privacy, equity, dehumanization, and decision-making dilemmas.

**Objective:**

We aim to critically analyze the main ethical implications of AI in end-of-life palliative care and examine the benefits and risks. We propose strategies for ethical and responsible implementation.

**Methods:**

We conducted an integrative review of studies published from 2020 to 2025 in English, Portuguese, and Spanish, identified through systematic searches in PubMed, Scopus, and Google Scholar. Inclusion criteria were studies addressing AI in palliative medicine focusing on ethical implications or patient experience. Two reviewers independently performed study selection and data extraction, resolving discrepancies by consensus. The quality of the papers was assessed using the Critical Appraisal Skills Programme checklist and the Hawker et al tool.

**Results:**

Six key themes emerged: (1) practical applications of AI, (2) communication and AI tools, (3) patient experience and humanization, (4) ethical implications, (5) quality of life perspectives, and (6) challenges and limitations. While AI shows promise for improving efficiency and personalization, consolidated real-world examples of efficiency and equity remain scarce. Key risks include algorithmic bias, cultural insensitivity, and the potential for reduced patient autonomy.

**Conclusions:**

AI can transform palliative care, but implementation must be patient-centered and ethically grounded. Robust policies are needed to ensure equity, privacy, and humanization. Future research should address data diversity, social determinants, and culturally sensitive approaches.

## Introduction

### Background

The review focused on studies published between 2020 and 2025 to capture the most recent advances in artificial intelligence (AI) technologies and their application in clinical practice, as the field has evolved rapidly in the last 5 years [1]. This approach ensures the relevance of findings to current and emerging ethical challenges.

AI is a subject within computer science that develops systems capable of performing tasks that simulate human capabilities, such as learning, reasoning, and decision-making. Machine learning (ML) allows algorithms to learn from data without explicit programming within this field. At the same time, deep learning, a subset of ML, uses advanced neural networks to analyze large volumes of information and generate accurate predictions.

AI significantly transforms the traditional health care paradigm toward an evidence-based and patient-centered model. Its application in areas such as the anticipation of complications, the personalization of treatments, and the optimization of resources has proven to be a key catalyst for improving the quality and efficiency of medical care [2].

Palliative medicine has also begun to benefit from the transformative potential of these technologies. This type of care, aimed at patients with advanced or terminal illnesses, seeks to alleviate physical, emotional, and spiritual suffering while seeking to improve quality of life and promote dignity in the final moments [3]. Palliative care encompasses a wide range of conditions, including advanced-stage oncological diseases (metastatic lung, breast, or pancreatic cancer) and nononcological illnesses such as neurodegenerative disorders (amyotrophic lateral sclerosis and late-stage Parkinson), end-stage organ failures (heart, lung, or renal disease), and severe respiratory conditions (chronic obstructive pulmonary disease). These patients, regardless of their specific diagnosis, share everyday needs: symptom relief, emotional support, and dignity preservation as they approach the end of life. The integration of AI in this sensitive context must, therefore, address the heterogeneity of these conditions while upholding ethical principles.

AI in palliative medicine includes tools, such as predictive models, to identify specific needs, wearable devices to monitor symptoms in real time, and virtual assistants that facilitate communication between patients, carers, and professionals. These innovations promise to improve clinical outcomes and enrich the patient experience by offering more personalized approaches [4]. However, its implementation poses significant ethical challenges due to the inherent vulnerability of patients and the complexity of end-of-life decisions. Thus, when we apply AI in palliative care, we must ensure that these tools do not reduce patients to mere actionable data but reinforce their humanity and dignity, honoring their individuality and right to compassionate and ethically informed care. Furthermore, it is crucial to consider how these technologies may affect human dignity and avoid a possible dehumanization of care [5]. In response to these concerns, various institutions have developed ethical guidelines to evaluate and regulate the responsible use of AI-based systems in sensitive contexts.

Despite enthusiasm for AI’s transformative potential, significant barriers to its widespread adoption in clinical practice remain. The lack of clear regulatory frameworks and consolidated examples of success highlights the urgent need for integrative research that addresses both the opportunities and the ethical and practical limitations of using AI in palliative care. As Miralles [2] points out in 2023, although multiple promising areas for applying AI in health care have been identified, few consolidated cases have achieved effective adoption in real clinical environments.

In recent years, various ethical self-assessment tools have been developed to verify the suitability of a system based on different ethical principles. In response to these concerns, various institutions have developed ethical guidelines to assess and regulate the responsible use of AI-based systems in sensitive contexts, such as the Ethical Guidelines for Trustworthy AI, the Draft Recommendation on the Ethics of Artificial Intelligence, and [6] the Barcelona Declaration [7]. Also, the AI Ethical Impact Group: From Principles to Practice [8], Technical Methods for Regulatory Inspection of Algorithmic Systems on Social Networking Platforms [9], or the Organisation for Economic Co-Operation and Development (OECD) of AI Systems Classification Framework [10], among others.

In line with international frameworks such as the Institute of Medicine (IOM) and the OECD, this review adopts a multidimensional understanding of “quality of care,” which includes safety, effectiveness, patient-centeredness, timeliness, efficiency, and equity as interrelated domains. While “efficiency” is thus an integral component of overall quality, for analytical clarity, we will at times refer to efficiency as system-level performance (resource optimization and process automation) and quality as patient-centered outcomes (dignity and symptom relief). This distinction, while recognizing their overlap, allows us to examine the specific effects of AI on both system operations and patient experience in palliative care.

### Theoretical Justification

Ethical reflection on palliative care and AI is rooted in classical and contemporary philosophy.

In his *Nicomachean Ethics* [11], Aristotle posits the notion of the “good life” as the realization of the highest human capacities through virtue, wisdom, and justice. From this perspective, a “good death” implies respecting the dignity and well-being of the patient even at the end of life.

For his part, Immanuel Kant, in the Foundation of the *Metaphysics of Morals* [12], argues that human dignity is an intrinsic and inalienable value, which precludes treating people as mere means to an end, even in medical or technological contexts; this requires that any intervention, including the application of AI, respects the autonomy and inherent value of each patient.

Finally, Emmanuel Lévinas, in *Totality and Infinity* [13], introduces the ethics of otherness, which stresses the importance of recognizing and preserving the uniqueness of the other. This approach is particularly relevant in palliative care, where care must focus on the individuality and dignity of the patient, avoiding technological reductionism that can depersonalize the end-of-life experience.

These 3 philosophical frameworks provide a sound basis for critically analyzing the opportunities and ethical challenges of integrating AI in palliative care.

We hypothesize that the application of AI in palliative medicine simultaneously offers significant opportunities for personalizing care and presents ethical risks that may compromise patient dignity. This study seeks to explore and examine this hypothesis through an integrative analysis of the recent literature.

### Objectives

1. We aim to examine current and potential applications of AI in palliative care: In this systematic review of the literature and recent cases, we will attempt to identify how AI is being used in end-of-life care, including tools for symptom management, clinical decision support, and communication between professionals, patients, and families.
2. To analyze the ethical implications of using AI in palliative care, we will investigate the ethical dilemmas arising from the integration of intelligent technologies in this field, such as privacy and handling of sensitive data, patient autonomy, equity in access to technology, and the possibility of depersonalizing care.
3. We aim to assess the impact of AI on patient experience and dignity at the end of life: To assess how the presence of AI influences the perception of quality of life, respect for dignity, and satisfaction of the emotional and spiritual needs of patients and their families.
4. We aim to propose recommendations for the ethical implementation of AI in palliative care.

## Methods

### Study Design

This research was carried out as an integrative review, which allows for synthesizing information from various study designs and offers a wide viewpoint on a challenging subject. The review included studies published in Spanish, Portuguese, and English between 2020 and January 2025. Due to their applicability in the biological and technological domains, the scientific databases consulted were PubMed, Scopus, and Google Scholar. Given the rapid development of the field over the past 5 years, we chose to focus on this recent period to ensure the inclusion of the most up-to-date and relevant advancements in AI applied to palliative medicine. This decision is supported by recent systematic reviews documenting a significant increase in the number and scope of published studies in this area [14].

### Search and Selection Process

The search strategy was designed to ensure a rigorous and systematic approach. Keywords such as “artificial intelligence,” “palliative care,” “palliative medicine,” “medical ethics,” “machine learning,” and related combinations were used (Multimedia Appendix 1). The search process followed the PRISMA (Preferred Reporting Items for Systematic Reviews and Meta-Analyses; Multimedia Appendix 2) guidelines, which provide a standardized framework for transparent and comprehensive reporting, including a 29-item checklist and a 4-phase flow diagram to document the identification, screening, eligibility, and inclusion of studies according to Page et al [15].

Study eligibility was assessed in 2 stages:

- Title and abstract screening: Two reviewers screened all papers and abstracts for relevance.
- Full-text review: The same reviewers independently assessed the full texts of potentially eligible studies.

Two reviewers independently conducted both the study selection and the quality assessment processes. Any discrepancies between reviewers were resolved through consensus discussions or, if necessary, by consulting a third reviewer.

Inclusion criteria were (1) studies published between 2020 and 2025, (2) research addressing the use of AI in palliative medicine, and (3) articles analyzing ethical implications or the patient experience in this context.

Exclusion criteria were (1) studies not explicitly focused on palliative medicine, (2) research lacking ethical analysis or patient experience analysis, and (3) duplicate or non–peer-reviewed publications.

The PRISMA flow diagram summarizes the study selection process, including the number of records identified, screened, excluded, and included at each stage.

### Data Extraction

Data extraction was performed independently by 2 reviewers (AGA and ASV). Any discrepancies were discussed and resolved collaboratively. Extracted data included study design, population, AI application, ethical focus, primary findings, and limitations.

### Quality Appraisal

The quality of the included studies was assessed using 2 tools to ensure methodological rigor. Two reviewers performed the quality assessment independently, with discrepancies resolved by consensus.

The Critical Appraisal Skills Programme (CASP) checklist [16] was applied as a complementary tool to further assess each study’s methodological quality and transparency (Table 1).

**Table 1:** Critical appraisal of included studies using CASP^a^ checklist.

Study area and author (year)		Key contribution	CASP rating
**Prediction and clinical decision-making**			
	Balch et al (2024) [17]	Review of AI^b^ for predicting PRO^c^ measures	Medium
	Strand et al (2024) [18]	AI/ML^d^ model to identify hospitalized patients with cancer needing palliative care	High
	He et al (2024) [19]	Effective in targeting palliative support	High
	Liu et al (2023) [20]	Accurate prediction of short-term mortality	Medium
	Heinzen et al (2023) [21]	Improved early referral to palliative care	High
	Morgan et al (2022) [22]	AI improved early identification	High
	Porter et al (2020) [23]	Critical reflection on risks or opportunities of AI prediction in palliative care	Medium
**Symptom management and quality of life**			
	Salama et al (2024) [24]	A systematic review of AI/ML in cancer pain management	High
	Lazris et al (2024) [25]	Comparison of AI-generated content (ChatGPT) vs NCCN^e^ guidelines for cancer symptoms	Medium
	Ott et al (2023) [26]	Impact of smart sensors on the “total care“ principle in palliative care	Medium
	Deutsch et al (2023) [27]	Improved monitoring and better symptom tracking	High
	Yang et al (2021) [28]	Wearables and ML to predict 7-day mortality in terminal cancer	Medium
**Communication and emotional support**			
	Gondode et al (2024) [29]	Performance of AI chatbots (ChatGPT vs Gemini) in palliative care education	Medium
	Srivastava and Srivastava (2023) [30]	GPT-3’s potential to improve palliative care communication	Medium
**Process automation and modeling**			
	Reason et al (2024) [31]	LLMs^f^ for automating economic modeling in health care	Low
	Kamdar et al (2020) [32]	Debate on AI’s future in palliative care or hospice (benefits vs risks)	Medium
	Windisch et al (2020) [3]	AI’s role in improving the timing or quality of palliative interventions	Medium
**Ethics and challenges**			
	See (2024) [33]	AI as an ethical advisor in clinical contexts	Medium
	Adegbesan et al (2024) [34]	Ethical challenges of AI integration in palliative care	High
	Ranard et al (2024) [35]	Minimizing algorithmic biases in critical care via AI	High
	De Panfilis et al (2023) [36]	Framework for future policy	High
	Ferrario et al (2023) [5]	Ethics of algorithmic prediction of end-of-life preferences	High
	Meier et al (2022) [37]	Framework for ethical algorithm-based clinical decisions	Medium
**Research and review of advances**			
	Bozkurt et al (2024) [1]	Protocol for assessing AI data diversity in palliative care	Medium
	Macheka et al (2024) [38]	Prospective assessment of AI in postdiagnostic cancer care. High feasibility and good patient feedback.	High
	Vu et al (2023) [14]	Systematic review of ML applications in palliative care	High
	Reddy et al (2023) [39]	Review of AI advances for palliative cancer care	Medium
	Barry et al (2023) [40]	Challenges for evidence-based palliative care delivery	Medium
	Chua et al (2021) [41]	Path to AI implementation in oncology; pragmatic roadmap created	Medium

^a^CASP: Critical Appraisal Skills Programme.

^b^AI: artificial intelligence.

^c^PRO: patient-reported outcome.

^d^ML: machine learning.

^e^NCCN: National Comprehensive Cancer Network.

^f^LLM: large language model.

The Hawker et al (2002) [42] checklist allows for systematic evaluation across diverse research designs. Each study was scored independently across 11 domains: clarity of purpose, study design, methodology, sampling, data analysis, ethical implications, relevance, transferability, results, discussion, and theoretical basis. Scores range from 1 (very poor) to 4 (good) for each domain; an overall quality score was calculated as the mean of all domain scores (Table 2).

**Table 2:** Methodological quality assessment of all included studies according to the criteria of Hawker et al [42]: 1=very poor, 2=poor, 3=fair, and 4=good.

Study	Clarity of purpose	Design	Methodology	Sampling	Analysis	Ethical implications	Relevance	Transferability	Results	Discussion	Theoretical basis	Overall quality
Balch et al [17]	4	4	4	2	3	3	3	4	4	3	3	3.27
Strand et al [18]	4	3	3	2	3	3	3	4	4	3	3	3.09
He et al [19]	2	3	3	2	3	3	3	4	4	3	3	2.91
Liu et al [20]	4	3	3	2	3	3	3	4	4	3	3	3.09
Heinzen et al [21]	4	3	3	2	3	3	3	4	4	3	3	3.09
Morgan et al [22]	4	3	2	3	3	3	3	4	4	3	3	3.09
Porter et al [23]	2	3	3	2	3	3	3	4	4	3	3	2.91
Salama et al [24]	4	3	3	2	3	3	3	4	4	3	3	3.09
Lazris et al [25]	4	3	3	2	3	3	3	4	4	3	3	3.09
Ott et al [26]	4	3	3	2	3	3	3	4	4	3	3	3.09
Deutsch et al [27]	2	3	3	2	3	3	3	4	4	3	3	2.91
Yang et al [28]	4	3	3	2	3	3	3	4	4	3	3	3.09
Gondode et al [29]	4	3	3	2	3	3	3	4	4	3	3	3.09
Srivastava and Srivastava [30]	4	3	3	2	3	3	3	4	4	3	3	3.09
Reason et al [31]	4	3	3	2	3	3	3	4	4	3	3	3.09
Kamdar et al [32]	2	3	3	2	3	3	3	4	4	3	3	2.91
Windisch et al [3]	4	3	3	2	3	3	3	4	4	3	3	3.09
See [33]	4	3	3	2	3	3	3	4	4	3	3	3.09
Adegbesan et al [34]	4	3	3	2	3	3	3	4	4	3	3	3.09
Ranard et al [35]	2	3	3	2	3	3	3	4	4	3	3	2.91
De Panfilis et al [36]	4	3	3	2	3	3	3	4	4	3	3	3.09
Ferrario et al [5]	4	3	3	2	3	3	3	4	4	3	3	3.09
Meier et al [37]	4	3	3	2	3	3	3	4	4	3	3	3.09
Bozkurt et al [1]	2	3	3	2	3	3	3	4	4	3	3	2.91
Macheka et al [38]	4	3	3	2	3	3	3	4	4	3	3	3.09
Vu et al [14]	4	3	3	2	3	3	3	4	4	3	3	3.09
Reddy et al [39]	4	3	3	2	3	3	3	4	4	3	3	3.09
Barry et al [40]	2	3	3	2	3	3	3	4	4	3	3	2.91
Chua et al [41]	4	3	3	2	3	3	3	4	4	3	3	3.09

### Thematic Analysis

Extracted data were thematically coded and grouped into 6 key categories—prediction and clinical decision-making, symptom management and quality of life, communication and emotional support, process automation and modeling, ethical implications, and research and review of advances in AI [14]—reflecting the main areas presented in the Results section.

### Ensuring Methodological Rigor

To maximize the reliability and validity of the review:

- Triangulation: findings were compared across studies to identify consistent patterns.
- Peer review: the methodology was reviewed using bioethics and AI.
- Critical evaluation: each study was assessed for quality, relevance, and validity using the criteria above.

This process allowed identifying strengths, limitations, and potential biases in the included studies [43].

## Results

### Overview

**Figure 1:**
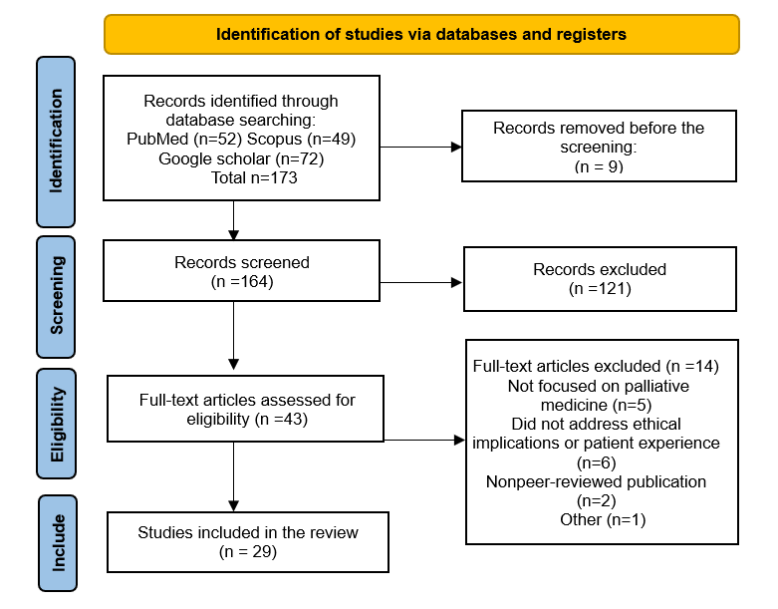
PRISMA 2020 flow diagram illustrating the study selection process. The diagram shows the number of records identified, duplicates removed, records screened, full-text papers assessed, and studies included in the review. Adapted from the PRISMA 2020 Statement [15].

This section presents a thematic synthesis of the main findings regarding the ethical and practical implications of AI in palliative medicine at the end of life. The 29 included studies, published between 2020 and January 2025, covered various clinical contexts, populations, and AI applications. The results are structured in 6 key areas identified in the literature (Figure 1).

The following tables provide a comprehensive synthesis of the 29 studies included in this review.

In Table 1, we present the ratings obtained for the methodological quality of each study according to the CASP checklist. Two independent reviewers rated each item as “yes,” “no,” or “unclear,” and the percentage of responses was calculated to assign an overall rating of high, medium, or low.

Table 2 shows the quality scores in 9 domains using the Hawker et al [42] instrument. Two reviewers rated each domain from 1 (very poor) to 4 (excellent), and we report both the individual domain scores and the overall mean quality score for each item.

Table 3 summarizes the key characteristics of the included studies. For each study, we list the author, year, country, design, AI/ML application, population or setting, study objective, and principal findings, and group them according to the 5 thematic areas identified in our review.

The analysis of the included studies revealed 6 key thematic areas in the application of AI in palliative care. These thematic areas provide a comprehensive overview of the current landscape and highlight both the opportunities and challenges presented by AI in this field.

**Table 3:** Characteristics of included studies. The main characteristics of the included studies are by thematic area, design, and key findings.

Author (year)	Country	Study design	AI^a^ application	Population or context	Study aim	Key findings
Balch et al (2024) [17]	United States	Review	AI for predicting PROs^b^	Patients with advanced cancer	Explore the use of AI in predicting PROs	AI shows potential but lacks validation
Strand et al (2024) [18]	United States	ML^c^ model development	Mortality prediction tool	Hospitalized patients with cancer	Develop a model to identify palliative needs	High predictive value for end-of-life care
He et al (2024) [19]	United States	Cohort study	ML for palliative consultation allocation	Patients with cancer	Assign consultations based on predicted need	Effective in targeting palliative support
Liu et al (2023) [20]	Taiwan	Observational	Wearables and ML	Patients with terminal cancer	Predict mortality risk in real time	Accurate prediction of short-term mortality
Heinzen et al (2023) [21]	Germany	RCT^d^	ML timing intervention	Primary care	Assess the impact on care timing	Improved early referral to palliative care
Morgan et al (2022) [22]	United States	RCT	AI prediction of care needs	Advanced cancer	Evaluate AI vs traditional triage	AI improved early identification
Porter et al (2020) [23]	United Kingdom	Critical reflection	Ethical analysis of prediction	General palliative care	Reflect on risks and values in AI prediction	Warns about the dehumanization risk
Salama et al (2024) [24]	United States	Systematic review	ML in pain management	Patients with cancer	Evaluate AI effectiveness in pain treatment	Supports the integration of AI tools
Lazris et al (2024) [25]	United States	Comparison study	ChatGPT vs NCCN^e^	Cancer symptom guidance	Evaluate content quality	AI is aligned with guidelines in most areas
Ott et al (2023) [21]	Germany	Observational	Smart sensors for monitoring	Palliative care patients	Assess the “total care” principle using technology	Improved monitoring and better symptom tracking
Deutsch et al (2023) [27]	Germany	Observational	ML for PROs monitoring	Metastatic cancer	Track patient outcomes	Improved reporting and early alerts
Yang et al (2021) [28]	China	Cohort study	Wearables and ML	Terminal cancer	Predict 7-day mortality	High predictive accuracy
Gondode et al (2024) [29]	United States	Comparative study	ChatGPT vs Gemini in education	Health care professionals	Compare chatbot effectiveness	Both tools are effective; Gemini is more accurate
Srivastava and Srivastava (2023) [30]	India	Exploratory	GPT-3 communication support	General palliative population	Examine AI in patient-clinician dialogue	Potential for improving conversations
Reason et al (2024) [31]	United Kingdom	Implementation study	LLMs^f^ for economic modeling	Palliative systems planning	Automate health economic models	LLMs reduce the workload but need oversight
Kamdar et al (2020) [32]	United States	Debate or commentary	AI in palliative care	General palliative systems	Debate AI’s pros and cons	Highlights opportunities and ethical risks
Windisch et al (2020) [3]	Germany	Case study	AI-enhanced timing	Hospital-based care	Improve intervention timing	Faster, more targeted responses
See (2024) [33]	United States	Qualitative	AI as an ethical advisor	Oncology settings	Evaluate AI-generated ethical suggestions	Useful but lacking nuance
Adegbesan et al (2024) [34]	Nigeria	Thematic analysis	Ethical challenges of AI	Low-resource settings	Explore equity and justice issues	AI raises equity concerns
Ranard et al (2024) [35]	United States	Technical study	Bias minimization algorithms	Critical care AI	Reduce bias in predictions	The algorithm reduced disparities in results
De Panfilis et al (2023) [36]	Italy	Conceptual framework	Ethical issues in AI	Palliative care context	Define ethical concerns	Framework for future policy
Ferrario et al (2023) [5]	United Kingdom	Ethical analysis	Predictive systems for end-of-life	Hospice settings	Assess algorithmic risks	Need for transparent systems
Meier et al (2022) [37]	United States	Framework proposal	AI clinical decision support	Advanced illness patients	Propose ethical guidance	Applicable for clinical protocol design
Bozkurt et al (2024) [1]	Turkey	Protocol	Diversity metrics in data	Mixed cancer cohorts	Establish a framework for diversity	Supports inclusive data use
Macheka et al (2024) [38]	Zimbabwe	Prospective evaluation	AI in postdiagnosis care	Rural patients with cancer	Evaluate implementation outcomes	High feasibility and good patient feedback
Vu et al (2023) [14]	Switzerland	Systematic review	ML in palliative care	Various populations	Map applications and outcomes	AI is growing in scope and evidence
Reddy et al (2023) [39]	India	Narrative review	AI for palliative oncology	Patients with cancer	Summarize recent AI use	Progress is seen, but fragmented evidence
Barry et al (2023) [40]	United States	Survey	Evidence-based palliative AI	Clinicians and patients	Identify barriers to adoption	Concerns about data quality and trust
Chua et al (2021) [41]	Singapore	Implementation framework	AI in oncology	Urban hospitals	Design path for AI adoption	Pragmatic roadmap created

^a^AI: artificial intelligence.

^b^PRO: patient-reported outcome.

^c^ML: machine learning.

^d^RCT: randomized controlled trial.

^e^NCCN: National Comprehensive Cancer Network.

^f^LLM: large language model.

### Prediction and Clinical Decision-Making

AI has demonstrated significant potential in supporting clinical decision-making and anticipating patient needs in palliative care. For example, Strand et al [18] developed a machine learning model that more accurately identified hospitalized patients with cancer who could benefit from specialized palliative care, outperforming traditional approaches. Similarly, Salama et al [24] and Liu et al [20] reported that AI tools can help personalize pain management and predict imminent terminal events, allowing for more timely interventions. However, Porter et al [23] cautioned that excessive reliance on algorithmic predictions, especially when models lack transparency or interpretability, may undermine human sensitivity and clinical judgment in complex palliative care scenarios.

### Symptom Management and Quality of Life

Effective symptom management and quality of life improvement are central in palliative medicine, and AI tools are being tested in oncological and nononcological populations. AI applications have facilitated individualized pain control and symptom monitoring for patients with cancer [24]. In noncancer contexts, Ott et al [26] described using smart sensors for real-time symptom tracking in neurodegenerative diseases. However, studies such as by Deutsch et al [27] highlighted the risk of bias when training datasets underrepresent specific populations (patients without cancer or minority groups), potentially limiting the generalizability and fairness of AI-driven symptom management.

### Communication and AI Tools

AI-based communication tools, including chatbots and natural language processing models, have been explored to support interactions between professionals, patients, and families. Gondode et al [29] and Srivastava and Srivastava [30] analyzed large language models GPT-3 to facilitate information delivery and emotional support. However, these tools often reflect Western bioethical principles. They may not adapt well to cultural contexts where family-centered decision-making or gradual truth disclosure is preferred [34]. Several studies reported that AI trained on Anglo-Saxon datasets may misinterpret emotional cues or cultural preferences, underlining the need for culturally sensitive models and community co-design.

### Process Automation and Modeling

AI process automation can optimize resource allocation and improve efficiency in palliative care. Reason et al [31] demonstrated how large language models can automate economic modeling, potentially reducing costs and improving service access. Windisch et al [3] emphasized the benefits of AI in improving the timing and quality of palliative interventions. However, Kamdar et al [32] stressed the importance of maintaining a patient-centered approach, even in highly automated environments.

### Ethical Implications

The ethical challenges of AI in palliative medicine are complex and multifaceted. Ferrario et al [5] analyzed the need for transparency and accountability in algorithmic prediction of end-of-life preferences. Ranard et al [35] addressed the risks of algorithmic bias, especially when models are trained on unrepresentative data, which can lead to inequitable care decisions. Finally, See [33] explored the potential of AI as an ethical advisor but noted that this application is still in its early stages and requires further research.

Regarding the ethical design of data-driven decision support tools, Bak et al [44] discuss the importance of considering ethical principles, such as algorithmic fairness and privacy, in the development of decision support tools in oncology, with direct implications for palliative medicine.

Furthermore, regarding the ethical design of data-driven decision support tools, Balch et al [17] discuss the importance of considering ethical principles, such as algorithmic fairness and privacy, in developing decision support tools in oncology, with direct implications for palliative medicine.

Ethical considerations should also be considered in cancer chatbots. In their study, Chow et al [45-52] address the need for transparency and informed consent in the use of AI-based chatbots in cancer care, emphasizing the risks of dehumanization and loss of trust.

### Research and Review of Advances in AI

Recent reviews and methodological studies have documented advances and limitations in AI applications for palliative care. Vu et al [14] systematically reviewed ML applications, highlighting the need for more robust, real-world evidence. Reddy et al [39] summarized advances in AI-based symptom management, and Bozkurt et al [1] developed protocols to assess the robustness of these applications. Macheka et al [38] evaluated the included role of AI in postdiagnostic treatment pathways, emphasizing the need for continuous research and ethical oversight.

AI offers innovative prediction, symptom management, communication, and resource optimization solutions in palliative medicine. However, its implementation is accompanied by significant ethical, cultural, and practical challenges, especially regarding equity, humanization, and respect for patient autonomy. The literature highlights the importance of addressing patient heterogeneity, cultural context, and social determinants to ensure that AI applications are practical and ethically acceptable.

## Discussion

### Principal Findings

This integrative review demonstrates that AI is increasingly present in palliative care, offering innovative solutions for clinical prediction, symptom management, communication, and process automation. The main findings suggest that while AI can improve efficiency and support decision-making, there remains a significant lack of consolidated, real-world examples that simultaneously demonstrate both efficiency and equity in outcomes. Most published studies focus on technical feasibility or operational improvements, but few document how AI enhances equitable access or patient-centered outcomes across diverse populations. This gap underscores the need for more robust, contextually grounded evidence to guide the ethical implementation of AI in end-of-life care [36].

A key finding is the persistent tension between efficiency and quality. Although frameworks such as the IOM and OECD recognize efficiency, equity, and patient-centeredness as embedded dimensions of quality, our review shows that improvements in system-level efficiency (resource optimization, automated symptom tracking) do not always translate into perceived improvements in care quality by patients and families. In palliative care, relational and dignity-centered outcomes, such as humanization, emotional support, and respect for autonomy, remain fundamental and may be at risk if AI is implemented without careful ethical consideration [36].

It should be noted that the paper distinguishes between “quality” of care and “efficiency.” In contrast, efficiency, equity, and patient-centeredness are internationally recognized as embedded measures of quality of care. According to frameworks such as the IOM [49], quality of care encompasses 6 interrelated domains: safety, effectiveness, patient-centeredness, timeliness, efficiency, and equity. Thus, efficiency is not a separate attribute but an integral component of quality, alongside equity and patient-centeredness. We recommend harmonizing the terminology and analysis to reflect this international consensus, avoiding an artificial dichotomy between quality and efficiency.

### Comparison to Prior Work

Our findings align with previous reviews, highlighting both AI’s transformative potential in palliative settings and the ethical challenges it introduces. Recent literature confirms that AI’s integration in palliative care is still early, with limited robust evidence for improved equity or patient experience. While some studies report promising advances in prediction and symptom management, others caution about algorithmic bias, lack of transparency, and the risk of dehumanization [50].

The review expands on prior work by addressing ethical principles’ historical and cultural variability. Many AI tools in palliative care are developed and validated in Western contexts, reflecting assumptions about autonomy, truth-telling, and individual decision-making that may not be universally applicable. Studies show that AI models trained on Anglo-Saxon datasets can misinterpret emotional cues or cultural preferences, particularly in Southern European, Latin American, or other non-Western settings where family-centered decision-making and gradual truth disclosure are everyday occurrences [34]. This highlights the need for culturally sensitive AI models and participatory design processes.

### Real Case: Mortality Prediction and Advance Care Planning

A recent study analyzed the implementation of an AI system designed to predict the likelihood of a patient dying in the next 12 months to facilitate timely discussions about palliative care. The study developed an explainable ML model using electronic health records data to proactively identify patients with advanced cancer at high risk of mortality. The model demonstrated strong predictive performance (area under receiver operating characteristic curve 0.861) and was intended to support early integration of palliative care in outpatient oncology settings [51]. However, introducing these predictions into the clinical process generated significant disagreements among health care professionals, patients, and family members. The main concerns included:

- Patient autonomy: algorithmic predictions were sometimes used without adequate informed consent, potentially undermining patients’ ability to decide about their care.
- Justice and equity: models trained on unrepresentative data risked introducing bias, disproportionately affecting marginalized groups.
- Beneficence and nonmaleficence: overreliance on AI predictions led to inappropriate interventions, such as premature end-of-life planning, without considering individual complexity.

This case highlights the need for a rigorous ethical approach to integrating AI into palliative care, ensuring that technologies complement clinical judgement and human empathy, not replace them.

### Strengths and Limitations

This integrative review has several strengths. First, it offers a comprehensive and up-to-date synthesis of the recent literature on AI in end-of-life palliative care, focusing on the period between 2020 and 2025. This time frame was chosen to reflect the most current technological developments and their ethical implications. Second, the review’s methodological rigor is reinforced using 2 quality appraisal tools, CASP and Hawker et al [42], which allowed a systematic evaluation across diverse study designs. Additionally, including philosophical, clinical, and ethical perspectives provides a multidimensional framework for understanding the challenges and opportunities of AI in this sensitive field. Furthermore, the review is enriched by presenting a real-world case, illustrating the practical dilemmas and tensions encountered when implementing predictive AI tools in clinical settings.

Nonetheless, several limitations must be acknowledged. The narrowed time frame may have excluded relevant earlier studies; however, this was a deliberate choice to focus on recent developments most applicable to current practice. The language scope was restricted to English, Portuguese, and Spanish, which may limit the generalizability of findings to other cultural contexts. Additionally, the heterogeneity of study designs and the variability in reporting standards made it difficult to perform a meta-analysis, which was replaced by thematic synthesis. Another limitation is that while some studies addressed social determinants of health (SDOH), such as literacy, socioeconomic status, or geographic inequalities, this aspect was not systematically captured across the entire corpus. Finally, given the fast-evolving nature of AI technologies, the findings and conclusions may become outdated over time, highlighting the need for ongoing surveillance and review.

The review of Ortiz et al [53] addressed the opportunities and challenges in using wearable sensors to monitor patients with cancer, including data integration and user acceptance issues.

### Future Directions

Integrating AI into palliative care necessitates a balanced approach that prioritizes ethical rigor, patient-centered outcomes, and cultural adaptability. Based on recent evidence, the following directions are critical for advancing this field responsibly.

### Enhancing Predictive Models With Diverse Data

ML models, such as those predicting mortality to facilitate early palliative care referrals in Medicare Advantage populations, demonstrate high accuracy but require broader validation across diverse demographics. Future research must prioritize datasets that include underrepresented groups (patients without cancer and ethnic minorities) to mitigate algorithmic bias and ensure equitable access to palliative services. For instance, models trained on SDOH and multi-institutional data could improve generalizability while addressing systemic disparities in end-of-life care [44].

### Ethical Co-Design of Decision Support Tools

The development of AI-driven decision support systems in oncology, as exemplified by the 4D PICTURE project, highlights the importance of participatory design involving patients, clinicians, and ethicists [54]. Key strategies include the following: (1) ethical review processes will need to be integrated to address data bias, privacy, and transparency; (2) ensuring AI tools align with cultural norms (family-centered decision-making in non-Western contexts); and (3) adopting frameworks such as design justice to empower marginalized voices in tool development.

### Hybrid Care Models Balancing Telehealth and Human Interaction

While telehealth improves accessibility and psychological comfort for palliative patients, studies emphasize that optimal care requires complementing AI tools with in-person interactions [54]. Future implementations should (1) integrate AI for routine monitoring (symptom tracking via wearables) while reserving complex decisions for clinician-patient dialogues and (2) address technical barriers (poor internet connectivity) that undermine interpersonal connection in telehealth.

### Transparency and Explainability in Mortality Prediction

Explainable AI models, such as the transparent mortality prediction tool for patients with cancer developed by Bertsimas et al [55], enhance clinician trust and facilitate shared decision-making. Recommendations include (1) standardizing reporting of model features (weight changes and albumin levels) to improve clinical interpretability and (2) validating tools in real-world settings to assess their impact on goals-of-care discussions and patient autonomy.

### AI as a Supplement, Not Replacement, for Human Judgment

Despite their reliability in answering breast cancer surgery queries (average score 3.98/5), AI chatbots such as ChatGPT must be framed as supplements to, not substitutes for, medical expertise [56]. Future work should (1) develop guardrails to prevent overreliance on AI, such as mandatory disclaimers urging users to consult clinicians, and (2) train chatbots to recognize cultural nuances in communication (gradual truth disclosure in specific populations).

### Ethical Deployment of Oncology Chatbots

Building on frameworks for human-centered AI in oncology [45], developers must prioritize (1) transparency: disclosing data sources and limitations to users, (2) autonomy: allowing patients to opt out of AI-driven interactions, and (3) equity: ensuring chatbots are accessible across literacy and socioeconomic levels.

### Conclusions

AI is poised to transform end-of-life palliative care, offering innovative clinical prediction, symptom management, communication, and resource optimization solutions. However, this review highlights that AI’s successful and ethical integration in such a sensitive field is far from straightforward.

First, while AI can improve efficiency and operational aspects of care, there is still a notable lack of consolidated, real-world examples demonstrating both efficiency and equity in outcomes. Most current evidence is derived from pilot studies or technical feasibility reports, with limited documentation of long-term, patient-centered benefits across diverse populations.

Second, the ethical challenges surrounding AI in palliative care are complex and multifaceted. Issues such as algorithmic bias, lack of transparency, risks to patient autonomy, and the potential for dehumanization must be addressed proactively. The review underscores that efficiency, equity, and patient-centeredness are not isolated goals but interrelated dimensions of quality care, as recognized by international frameworks such as the IOM.

Third, cultural context and patient heterogeneity are critical factors. Many AI tools are developed based on Western bioethical assumptions, which may not be universally applicable, especially in settings where family-centered decision-making or culturally nuanced understandings of pain and suffering prevail. Addressing SDOH and ensuring the inclusion of marginalized voices in AI development is essential for equitable implementation.

Fourth, patients’ and families’ experiences must remain at the heart of palliative care innovation. AI should complement, not replace, clinical judgment, human empathy, and the relational aspects that define quality end-of-life care.

### Key Recommendations

Our key recommendations are to (1) develop clear policies and regulatory frameworks to ensure fairness, privacy, transparency, and accountability in using AI in palliative care; (2) promote the humanization of care, design AI tools that support, rather than supplant, compassionate human interaction and shared decision-making; (3) foster continuous, multidisciplinary research to rigorously evaluate the benefits and risks of AI, address algorithmic biases, and adapt tools to diverse clinical and cultural contexts; and (4) encourage participatory approaches involving patients, families, clinicians, ethicists, and community representatives in designing, implementing, and evaluating AI systems.

### Final Perspective

Ultimately, the ethical adoption of AI in palliative medicine requires a careful balance between technological innovation and the preservation of human dignity. Only through patient-centered, culturally sensitive, and ethically grounded strategies can we maximize the benefits of AI while mitigating its risks, thus improving the experience and outcomes for patients and families at the end of life.

## Appendix

Multimedia Appendix 1 Review strategy and quality assessment.

Multimedia Appendix 2 PRISMA checklist.

## Abbreviations

AI: artificial intelligence
CASP: Critical Appraisal Skills Programme
IOM: Institute of Medicine
ML: machine learning
OECD: Organisation for Economic Co-Operation and Development
PRISMA: Preferred Reporting Items for Systematic Reviews and Meta-Analyses
SDOH: social determinants of health
