# Supplementary material for: Ethical Challenges and Opportunities of AI in End-of-Life Palliative Care: Integrative Review
Source: Interact J Med Res. 2025 May 14;14:e73517. doi: 10.2196/73517 (PMC12120364; doi:10.2196/73517)
Supplement: Multimedia Appendix 1 [file ijmr_v14i1e73517_app1.docx]

**Method for Table 1**

**Critical Appraisal (CASP Checklist)**

**Objective:** We assessed the methodological quality of the 29 included studies.

**Instrument:** We applied the CASP checklist appropriate to each study’s design:

- **CASP Qualitative Studies Checklist** (10 items)
- **CASP Randomised Controlled Trials Checklist** (11 items)
- **CASP Cohort Studies Checklist** (12 items)

*Note: We used only the items relevant to each study’s design.*

**Procedure:**

1. Two independent reviewers scored each item as “Yes”, “No” or “Unclear.”
2. We calculated the percentage of “Yes” responses out of the total applicable items for each study.
3. We assigned an overall quality rating based on that percentage:
   - **High**: > 80 % “Yes”
   - **Medium**: 50–80 % “Yes”
   - **Low**: < 50 % “Yes”
   - **Outcome:** This table shows each study’s High/Medium/Low rating.

**Method for Table 2**

**Methodological Quality (Hawker et al.)**

**Objective:** We generated a uniform quality score for all 29 quantitative or qualitative studies.

**Instrument:** We used Hawker et al.’s nine-domain appraisal tool:

1. Clarity of purpose
2. Study design
3. Methodology
4. Sampling
5. Data analysis
6. Ethical considerations
7. Relevance
8. Transferability
9. Discussion and theoretical basis

**Procedure:**

- Two independent reviewers scored each domain from 1 (very poor) to 4 (excellent).
- We calculated the mean of the nine domain scores for each article.
- We treated that continuous mean as the study’s overall quality indicator.

**Outcome:** This table lists every study’s nine domain scores and mean overall quality score.

**Method for Table 3**

**Characteristics of Included Studies**

**Objective:** We summarised the key attributes of the 29 included studies.

**Data fields extracted:**

- Author, year and country
- Study design
- Type of AI/ML application
- Population or context
- Study aim
- Principal findings

**Procedure:**

1. Two independent reviewers completed a standardised extraction form for each study.
2. We resolved any discrepancies by consensus.
3. We allocated each study to one of the five thematic areas we had identified.

**Outcome:** Table 3 presents the thematic grouping, study design and main findings for all 29 studies.
